# Tryptophan metabolism, disposition and utilization in pregnancy

**DOI:** 10.1042/BSR20150197

**Published:** 2015-10-30

**Authors:** Abdulla A.-B. Badawy

**Affiliations:** * School of Health Sciences, Cardiff Metropolitan University, Western Avenue, Cardiff CF5 2YB, Wales, U.K.

**Keywords:** albumin, free tryptophan, immunosuppression, kynurenine metabolites, non-esterified fatty acids, pre-eclampsia

## Abstract

Tryptophan (Trp) requirements in pregnancy are several-fold: (1) the need for increased protein synthesis by mother and for fetal growth and development; (2) serotonin (5-HT) for signalling pathways; (3) kynurenic acid (KA) for neuronal protection; (4) quinolinic acid (QA) for NAD^+^ synthesis (5) other kynurenines (Ks) for suppressing fetal rejection. These goals could not be achieved if maternal plasma [Trp] is depleted. Although plasma total (free + albumin-bound) Trp is decreased in pregnancy, free Trp is elevated. The above requirements are best expressed in terms of a Trp utilization concept. Briefly, Trp is utilized as follows: (1) In early and mid-pregnancy, emphasis is on increased maternal Trp availability to meet the demand for protein synthesis and fetal development, most probably mediated by maternal liver Trp 2,3-dioxygenase (TDO) inhibition by progesterone and oestrogens. (2) In mid- and late pregnancy, Trp availability is maintained and enhanced by the release of albumin-bound Trp by albumin depletion and non-esterified fatty acid (NEFA) elevation, leading to increased flux of Trp down the K pathway to elevate immunosuppressive Ks. An excessive release of free Trp could undermine pregnancy by abolishing T-cell suppression by Ks. Detailed assessment of parameters of Trp metabolism and disposition and related measures (free and total Trp, albumin, NEFA, K and its metabolites and pro- and anti-inflammatory cytokines in maternal blood and, where appropriate, placental and fetal material) in normal and abnormal pregnancies may establish missing gaps in our knowledge of the Trp status in pregnancy and help identify appropriate intervention strategies.

## Abstract (precis)

The essential amino acid tryptophan (Trp) is especially important in pregnancy for protein synthesis by mother and fetus, fetal growth and development and prevention of fetal rejection by immunosuppression. A Trp utilization concept based on these physiological requirements in pregnancy is proposed.

## INTRODUCTION

The essential amino acid L-tryptophan (Trp) is particularly important in pregnancy, because of the increased demand for maternal protein synthesis and fetal requirements for growth and development. It would therefore be physiologically disadvantageous if maternal Trp is depleted in pregnancy. Munn et al. [1] proposed that fetal rejection is prevented by suppression of T-cell activity through Trp depletion after cytokine induction of the extrahepatic Trp-degrading enzyme indoleamine 2,3-dioxygenase (IDO). The then prevailing view that Trp depletion is responsible for the anti-proliferative, anti-bacterial and anti-parasitic activity of the IDO inducer interferon-γ (IFN-γ) [2,3] led the above authors to apply the same principle to immunity against fetal rejection. However, immunosuppression in the presence of Trp deficiency cannot guarantee a successful pregnancy, given the above Trp requirements. Moreover, the experimental design of the depletion concept, aspects of the Trp status in pregnancy known at the time and the subsequent demonstration of the immunosuppressive properties of some Trp metabolites of the kynurenine (K) pathway (KP) strongly suggest the need to revise the depletion concept to one that reconciles the need for immunosuppression with that for Trp. In the present article, Trp metabolism and disposition in pregnancy will be reviewed and a Trp utilization concept fulfilling the above requirements will be discussed. Important questions regarding the interaction of Trp and its K metabolites with the immune system in pregnancy need to be addressed and it is hoped that the present review will stimulate further research that could contribute to improved understanding of issues in management of pregnancy and its complications.

## TRYPTOPHAN METABOLISM AND DISPOSITION

### Overview

In addition to proteins, Trp is the precursor of many physiologically important metabolites (Table 1). Only ≤1% of dietary Trp is used for protein synthesis, as the amount synthesized compensates for that degraded [4]. Most dietary Trp is therefore metabolized, via four pathways (Table 1), the quantitatively most important of which is the hepatic (oxidative) KP, accounting for >95% of Trp degradation [4]. Trp 2,3-dioxygenase (TDO; formerly Trp pyrrolase) is the first and rate-limiting enzyme of this pathway. TDO activity is increased after induction by glucocorticoids and activation by the Trp substrate or the cofactor haem and inhibited allosterically by an NAD (P) H-mediated feedback mechanism [4,5] and by progesterone and oestrogens [6].

**Table 1:** The Trp metabolic pathways and their main products

Pathway	Main products	Main function(s)
1 Decarboxylation	Tryptamine	Neuroactive amine
2 Transamination	Indolepyruvic acid	Anxiolytic, improves sleep, precursor of KA
3 Hydroxylation	5-HT (brain)	Behavioural control, cognition, emotion, mood, signalling pathways
	Melatonin (pineal)	Antioxidant, neuroprotection, circadian rhythm regulation
4 Oxidation	K	Precursor of Ks
	KA	Anxiolytic, neuroprotective, NMDA receptor antagonist; α-7 nicotinic acetylcholine receptor antagonist
	Anthranilic acid	Immune modulation
	3-HK	T-cell suppression
	Xanthurenic acid	Insulin binding
	3-HAA	T-cell suppression
	Nicotinic acid	Pellagra-preventing factor (vitamin B_3_)
	QA	NAD^+^ precursor, excitotoxic, NMDA receptor agonist
	Picolinic acid	Zinc binding
	NAD^+^(P)^+^(H)	Redox cofactors, cell viability and DNA repair

The KP is outlined in Figure 1. K is metabolized mainly by K hydroxylase (K mono-oxygenase) to 3-hydroxykynurenine (3-HK), which is then hydrolysed by kynureninase to 3-hydroxyanthranilic acid (3-HAA). At the acroleyl aminofumarate step, the pathway favours non-enzymic cyclization to quinolinic acid (QA), which proceeds to form nicotinic acid and then NAD^+^. Transamination of K and 3-HK is physiologically a minor reaction, because of the high *k*_m_ of K aminotransferase for its two substrates, but can be enhanced if [K] is increased, e.g. by Trp or K loading or if activity of K hydroxylase or kynureninase is inhibited [4,5].

**Figure 1:**
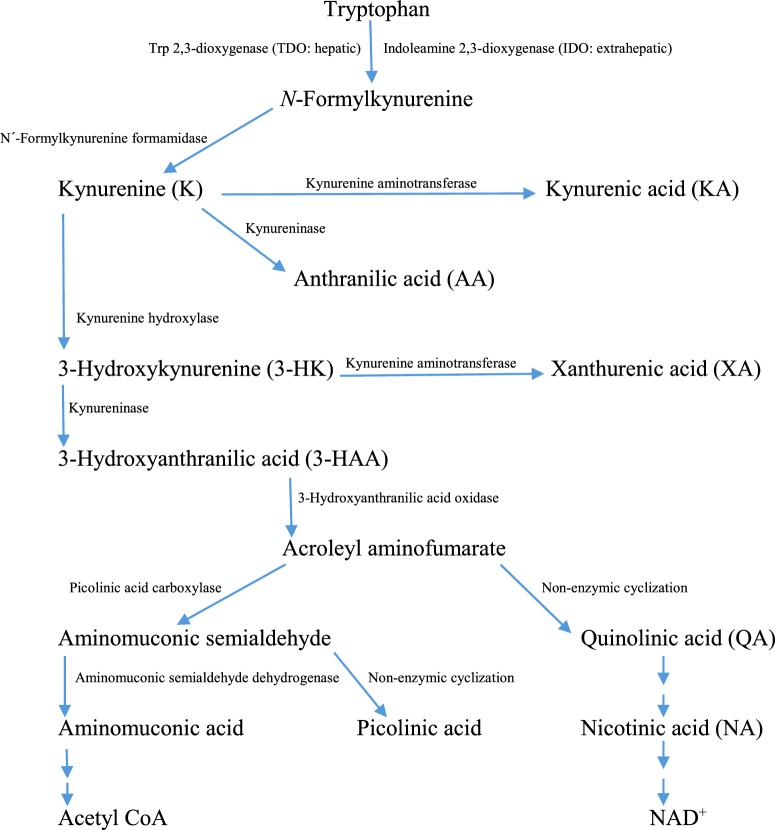
Schematic representation of the hepatic K pathway of Trp degradation Adapted from Figure 1 in [135]: Badawy, A.A.-B. (2014) Pellagra and alcoholism: a biochemical perspective. Alcohol Alcohol. **49**, 238–250.

The KP also exists extrahepatically, although only the hepatic pathway includes all the enzymes required for nicotinic acid synthesis. The extrahepatic pathway is controlled by another haemoprotein, IDO, the combined human tissue activity of which is normally, however only 5%–15% of that of hepatic TDO [7], but can be dramatically increased after immune activation by its principal effector IFN-γ [2]. The most remarkable feature of IDO is the astounding degree of its induction by IFN-γ and agents acting through it under a variety of pathological conditions, which ranges between 20- and 4000-fold, rendering it the major player in the control of Trp availability under such conditions. The placenta is the richest source of IDO [8]. Production of K metabolites is achieved normally by the flux of Trp down the hepatic pathway, but can be increased if TDO activity is enhanced. The type of metabolite formed extrahepatically depends on the enzymes present. As will be described below, TDO is also expressed extrahepatically in endometrium, embryonic and fetal tissues. It is also important to note that free Trp (the non-albumin-bound 5%–10% of circulating Trp that is immediately available for tissue uptake) determines to a large degree the hepatic uptake and subsequent flux of Trp down the KP [9].

### Regulation of tryptophan 2,3-dioxygenase and indoleamine 2,3-dioxygenase

TDO exists in livers of humans, rats, mice and some, but not all, other animal species in two forms: the active haem-containing holoenzyme and the inactive haem-free apoenzyme in a proportion of ∼1:1 [10]. The TDO haem saturation in these species is therefore ∼0.5 as a ratio or ∼50% as a percentage. Some other species, e.g. cat, frog, gerbil, golden hamster, guinea pig, ox, rabbit and sheep, lack the free TDO apoenzyme and the glucocorticoid induction mechanism and are sensitive to the toxicity of excess Trp [10]. Apart from the gerbil, they have lower free and total [Trp] than rats and mice [10]. These TDO-deficient species are therefore unsuitable as animal models of Trp-related human diseases. Also, because of mouse strain differences in Trp metabolism (see below), I believe that the rat is the animal model of choice in Trp-related studies. From the data in the study conducted by Badawy and Evans [10], it is apparent that rat and mouse plasma free and total [Trp] are at least 50% higher than those in human plasma. It could be argued that these higher Trp values reflect IDO inhibition by nitric oxide [11], whose levels are higher in rodents than humans [12]. However, a comparison of small intestinal IDO activities in two species in each of the above two groups (rats and mice with high and comparable plasma [Trp] and guinea pig and rabbit with low and comparable plasma [Trp], [10]) showed [13] that the rat IDO activity was 24-fold higher than that of mouse (73.01 compared with 3.02 nmol/min/g tissue) and that IDO activities in guinea pig and rabbit were 12.0 and 16.2 nmol/min/g of tissue. It is therefore unlikely that the plasma Trp differences are related to IDO activity. As stated above, the hepatic K pathway is responsible for ∼95% of total body Trp oxidation and hence plasma Trp availability under normal conditions. This is best exemplified by the observation [14] that deletion of the mouse TDO gene elevates plasma total [Trp] by 9.3-fold. An opportunity of establishing the relative contribution of IDO1 gene deletion to plasma Trp availability was not taken [15].

TDO is regulated by at least four mechanisms: glucocorticoid induction involving *de novo* enzyme synthesis, substrate activation and stabilization by Trp, cofactor activation by haem and feedback inhibition by NAD(P)H [4,5]. Transcription of the TDO gene by glucocorticoids is potentiated by glucagon, but inhibited by insulin and adrenaline [16]. Haem also appears to regulate the glucocorticoid induction and gene expression of TDO [17]. In relation to pregnancy, rat liver TDO is inhibited by both progesterone and oestrogens by a combination of two actions: prevention of conjugation of the apoenzyme with haem and inactivation of the holoenzyme [6]. In contrast with liver TDO, the extrahepatic IDO is fully haem-saturated. Its principal effector is IFN-γ [2]. Rat intestinal IDO activity is not induced by glucocorticoids, but is enhanced by Trp by 50% [18], compared with the several-fold enhancement of liver TDO.

### Plasma tryptophan disposition

As stated above, the small fraction (5%–10%) of circulating Trp that is not albumin-bound is freely available for uptake by organs and tissues. Free Trp is a labile parameter, the concentration of which can be influenced by hormonal, metabolic, nutritional and pharmacological factors [19]. Methodological pitfalls can also influence accuracy of free Trp determination. In particular, only freshly isolated plasma (or serum) should be ultrafiltered to prevent increased albumin binding after frozen storage [19]. Accurate interpretation of changes in plasma Trp disposition (Table 2) requires measurements of free and total [Trp] in the first instance, followed, if necessary, by those of the two determinants of binding, namely albumin and the physiological displacers of albumin-bound Trp, non-esterified fatty acids (NEFA). Note should also be taken of any prescribed or over-the-counter medication which may influence this binding, e.g. salicylate [the active moiety of the acetylsalicylate (aspirin) molecule] or acute intake of alcohol or of methylxanthines-containing soft drinks and hot beverages, such as tea, coffee and cocoa [19]. TDO or IDO induction requires demonstration of proportionate decreases in both free and total [Trp] with no change in Trp binding (expressed as the percentage free Trp). It is important to note here that the decreases in plasma free and/or total Trp following glucocorticoid induction of TDO and cytokine induction of IDO rarely exceed 30% ([7] and references cited therein). TDO inhibition should, by contrast, be associated with proportionate increases in both free and total [Trp] (usually of 20%–33%), without any change in Trp binding. Thus, it is not sufficient to conclude that TDO or IDO is induced based only on a decrease in total [Trp]. Free Trp determination is important not only for accurate interpretation of changes in Trp disposition, but also in assessing the baseline Trp metabolic status and its biological determinants [19]. As will be shown below, Trp-metabolic studies in relation to immune function in whole animals or humans have so far failed to include free Trp determination. Whereas measurement of free Trp may not be feasible or meaningful in immune-related studies *in vitro* using peripheral blood and other cell lines in culture, Trp binding and disposition in intact animals or humans requires assessment of free Trp.

**Table 2:** Plasma Trp disposition

Parameter	Change	Mechanism	Examples of effectors
Free Trp	Decrease	TDO/IDO induction	Glucocorticoids/IFN-γ
		Inhibition of lipolysis	Insulin, nicotinic acid, antilipolytic agents
	Increase	TDO inhibition	glucose, nicotinamide, anti-depressants
		Displacement from albumin	NEFA, catecholamines, ethanol, salicylate
		Decreased albumin	Pregnancy, liver diseases
Total Trp	Decrease	TDO/IDO induction	Glucocorticoids/IFN-γ
	Increase	TDO inhibition	glucose, nicotinamide, anti-depressants
% Free Trp	Unaltered	TDO/IDO induction	
		TDO inhibition	
	Decrease	Increased albumin binding	
	Increase	Decreased albumin binding	

For details, see previous studies [7,19].

## EXPERIMENTAL DESIGN OF THE TRYPTOPHAN DEPLETION CONCEPT IN PREGNANCY

The concept [1] draws on two observations: (1) expression of IDO by human syncytiotrophoblast cells; (2) decreased human maternal plasma total [Trp]. The experimental design is based on the ability of the IDO inhibitor 1-methyltryptophan (1-MT) to induce rapid T-cell rejection of all allogeneic concepti in pregnant mice. A number of issues arise from the design, most importantly is that it has been known for some considerable time that pregnancy is associated with elevation of maternal plasma free [Trp]. The design also did not involve assessment of Trp disposition or take into account the unusual Trp metabolic status and the greater immune sensitivity of (male), C57BL/6J, with which female CBA, mice were mated, nor could the authors have predicted that 1-MT will subsequently be shown to inhibit placental Trp transport. The Trp disposition status in pregnancy will be considered separately below and the following are brief accounts of aspects of the concept design:

### Tryptophan metabolism in C57BL/6J mice

Mouse strain differences in Trp metabolism could affect the study outcome. The C57BL/6J strain (hereafter referred to as C57) has the highest basal TDO activity and a consequently lower serum-free and total [Trp], resulting in decreased cerebral serotonin (5-HT) synthesis and turnover, which may be related to its alcohol preference [20–22]. Although male C57 mice show a greater response to glucocorticoid induction of TDO [20,23], their enzyme is impervious to activation by NEFA after acute ethanol administration [21]. It follows therefore that a NEFA elevation in C57 could result in accumulation of (free) Trp. Female C57 mice have a higher basal TDO activity, but a lesser response to cortisol, than males [20] and their plasma [Trp] is therefore likely to be even lower than in males. These C57-specific features could render this strain vulnerable.

### Immune sensitivity of the C57BL/6J mouse strain

Such vulnerability is of an immunological nature. The C57 appears to be particularly sensitive to immune insults. Thus, compared with BALBc, C57 mouse macrophages exhibit a greater response to lipopolysaccharide stimulation and a higher phagocytic capacity [24]. C57 mice also exhibit a greater sensitivity to liver injury caused by acute *Trypanosoma cruzi* infection [25] and to profound immunodeficiency after LP-BM5 retroviral infection [26].

### Inhibition of tryptophan transport by 1-methyltryptophan

Subsequent to publication of the depletion concept [1], the IDO inhibitor 1-MT was shown to inhibit Trp transport in human placenta [27] and fibroblast cell lines [28]. It is possible that this effect coupled with the lower plasma [Trp] in C57 mice could have further undermined Trp availability to concepti in the Munn et al. [1] study. The potential impact of the above C57-specific metabolic and immune features on the fate of syngeneic C57XC57 concepti exposed to 1-MT has not been tested. Whereas it is unlikely that 1-MT will induce fetal rejection, it may cause adverse effects. For example, deletion of the IDO gene from C57BL/6 mice (an alternative approach to 1-MT inhibition of IDO) induces a significant fetal weight loss, but does not affect fecundity (litter size) [15]. Also, the IDO inhibitory property of 1-MT is at odds with the previous observation [29] that it actually up-regulates IDO1 in human cancer cells.

### Human maternal plasma tryptophan is not depleted

Contrary to the concept [1], maternal plasma Trp is not depleted. The widely reported [30–39] decrease in late pregnancy applies to total (i.e. free + albumin-bound) Trp, whereas, when measured, free Trp is actually increased in late pregnancy. Normal fasting plasma free and total [Trp] in 60 healthy non-pregnant female USA subjects are 5.96±0.35 and 58±2 μM respectively (means ± S.E.M.), corresponding to 1.22±0.08 and 11.84±0.41 μg/ml respectively, with a percentage free Trp of 10.28 (A. A.-B. Badawy and D.M. Dougherty, unpublished work). In a further 25 female subjects, we observed values not dissimilar to the above ones (5.80±0.32 μM, 60±4 μM and 9.67±0.56% respectively). As shown in Table 3, plasma total [Trp] is decreased in all studies. Not all studies included a control (non-pregnant women) group, but, when controls were included, the decreases in total [Trp] ranged between 25% and 50%. With the exception of study by Tricklebank et al. [30], total [Trp] values in pregnancy ranged between 25 and 47 μM. Only six studies reported free [Trp] values, which were not decreased, thus excluding TDO or IDO induction (see above). The free Trp values ranged between 5.4 and 13.7 μM, resulting in a percentage free Trp of 18%–30%, compared with the 10% normal value observed in our studies. This increase in the percentage free Trp indicates decreased binding to albumin. Because of the rapid equilibration between the free and bound Trp fractions, the apparently normal free Trp values should be considered higher than would be expected physiologically. In addition, the apparently normal free Trp values in two of these six studies should have been higher, because subjects received food [32], which can induce an insulin-mediated lowering of free [Trp] [19] and a fluorimetric analytical procedure that causes loss of free Trp was used [33] rather than the modification [40] aimed at correcting this loss. Although the free [Trp] value in one study [30] was high, it could have been even-higher, if the authors prepared the ultrafiltrates from fresh and not frozen plasma (see above).

**Table 3:** Plasma Trp disposition in pregnancy: previous studies Values are micromolar means or percentages. Because of the absence of control groups in four of the above studies, historical control values for free Trp (5.96 μM) total Trp (58 μM) and the percentage free Trp (12%) are included in the present study for approximate comparison (see the text). Note that control total [Trp] values averaged for the other six studies (58 μM) are similar to our historical controls. *Not determined. For references, see the text.

Reference	Total Trp		Free Trp		Percentage free Trp	
	Control	Pregnant	Control	Pregnant	Control	Pregnant
[30]	*	53	*	12.2	*	23
[31]	65	46	10.0	13.7	15	30
[32]	58	29	7.3	5.4	12	19
[33]	*	31	*	6.4	*	21
[34]	*	40	*	7.5	*	19
[35]	*	35	*	6.3	*	18
[36]	54	40	*	*	*	*
[37]	48	25	*	*	*	*
[38]	72	47	*	*	*	*
[39]	53	33	*	*	*	*

The increase in free [Trp] is most probably due to decreased [albumin] and increased [NEFA]. Thus, pregnancy is associated with decreased circulating [albumin] [41], which undergoes gradual decreases with progress of normal pregnancy, with average (and range) values (in g/l) of 41 (31–51), 35.5 (26–45) and 32.5 (23–42) during the first, second and third trimesters respectively, compared with control non-pregnancy values of 47 (41–53) g/l [42]. The average decreases in [albumin] over the three trimesters are therefore 13%, 24% and 31%. As will be stated below, Trp binding to albumin is decreased when [albumin] drops by 19%. Thus, an albumin-related increase in free [Trp] can be expected to occur in the second and third trimesters, but not earlier. NEFA, the physiological displacers of albumin-bound Trp, are increased in late pregnancy [43–46]. Thus plasma [NEFA] are increased from control non-pregnancy levels by ∼20% during the first two and by a 44% maximum during the third trimester [46]. The combined effect of the albumin decrease and the NEFA increase in late pregnancy is likely to achieve the maximal elevation of free [Trp] (see below).

### Pregnancy in rats as a model of human pregnancy

Pregnancy in rats can be considered an accurate model of human pregnancy, as the changes in Trp metabolism and disposition in the former [6], illustrated here in Figure 2, reflect the aspects discussed above. Thus, in early and mid-pregnancy (up to day 12) in rats, both free and total [Trp] are proportionately elevated, with no change in Trp binding (expressed as the percentage free Trp), because of inhibition of hepatic TDO activity by progesterone and oestrogens. As discussed previously ([6] and referenced cited therein), oestrogens inhibit TDO activity in rats and humans, in contrast with the earlier reported enhancement in intact, but not adrenalectomized, rats, which is actinomycin-D-sensitive and therefore possibly glucocorticoid-mediated. Maximal elevations of free and total [Trp] in pregnant rats were 31% and 25% on days 8–12, coinciding with the maximal TDO inhibition on days 8–9 [6]. When TDO activity returned to normal control values on day 16, total [Trp] also returned to normal. However, free [Trp] remained elevated on day 16 (by 36%). As [NEFA] was not significantly altered on day 16, this increase in free Trp can be attributed to the 19% decrease in [albumin]. The 13% decrease in [albumin] on day 12 is presumably insufficient to influence Trp binding. On day 20, free [Trp] was hugely elevated (by 122%), presumably because of the continued decrease in [albumin] (21%) and, more importantly, the 76% increase in [NEFA]. The 21% decrease in total [Trp] on day 20 can be explained by increased tissue uptake of free Trp and the rapid equilibration between the free and albumin-bound fractions [19]. This rat model may provide an explanation of why plasma total [Trp] is decreased in late pregnancy in humans and illustrates the need to include plasma-free Trp determination in future studies in humans and whole animals. Notwithstanding the rapid equilibration between free and albumin-bound Trp, plasma free and total [Trp] values at any time represent the steady-state of Trp binding and disposition and do not reflect any methodological limitation, especially in view of the widely reported consequences of changes in Trp binding on tissue Trp uptake and further metabolism under both acute and chronic conditions. I therefore urge the research community, especially those in the immunology field, to consider, where appropriate, measuring plasma free as well as total [Trp] in humans and intact animal models in Trp-related studies.

**Figure 2:**
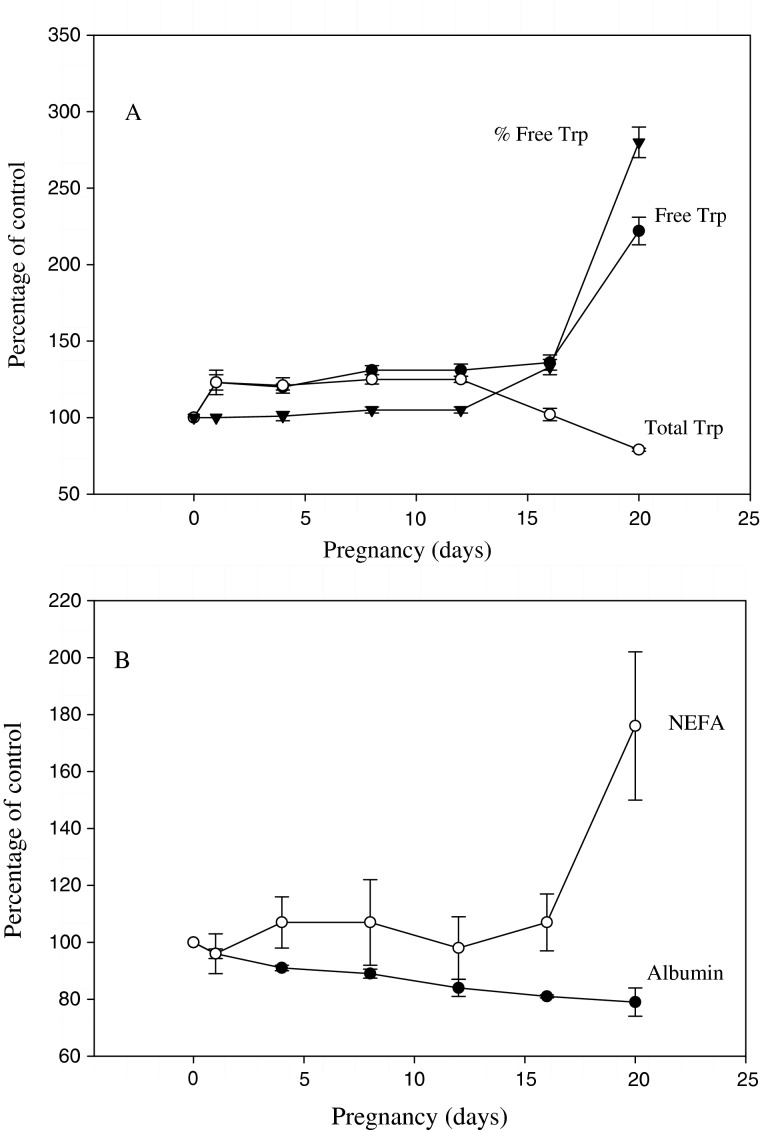
Effects of pregnancy in rats on serum free and total Trp concentrations and binding (A) and serum albumin and NEFA concentrations (B) Reproduced with kind permission from Obstetrics and Gynecology Science from [48]: Badawy, A.A.-B. (2014) Review: the tryptophan utilization concept in pregnancy. Obstet. Gynecol. Sci. **57**, 249–259; data in Table 1 from [6]: Badawy, A.A.-B. (1988) Effects of pregnancy on tryptophan metabolism and disposition in the rat. Biochem. J. **255**, 369–372.

In human pregnancy, maternal total Trp is also elevated in early pregnancy, returning to slightly below normal in mid-, before decreasing further in late, pregnancy [36], by 25%, a value comparable to the 21% decrease in rats. Thus, these changes in total [Trp] are mirrored by those observed in rats [6] and suggest that TDO activity may also be inhibited in early to mid-pregnancy in humans. Earlier studies in pregnant rats have suggested that TDO may be enhanced by oestrogens. However, subsequent studies in both rats and humans have clearly established the TDO inhibitory effects of both oestrogens and progesterone (see [6] for references). Human liver TDO (assessed in biopsy specimens) is inducible by cortisol [47] and although maternal plasma cortisol also rises in late pregnancy [42], the absence of a decrease in free [Trp] suggests that TDO activity is not elevated, possibly because of a potential inhibition by progesterone and oestrogens.

## IMPORTANCE OF TRYPTOPHAN AND ITS UTILIZATION FOR A SUCCESSFUL PREGNANCY

Trp depletion is physiologically disadvantageous to both mother and fetus, as they need Trp for many purposes [48]: (1) increased demand for protein synthesis by mother and fetus; (2) 5-HT synthesis for signalling pathways; (3) kynurenic acid (KA) for neuronal protection; (4) QA for NAD^+^ synthesis. None of these processes is achievable optimally if Trp is depleted in mother or fetus. A Trp utilization concept in pregnancy has been proposed [48] that provides the basis for fulfilling these physiological requirements and the following discussion provides data supporting and expanding this concept.

### Increased tryptophan requirement by the fetus

Protein and amino acid requirements [49] and transport [50] are increased in pregnancy. A comparison of maternal and umbilical cord [Trp] illustrates the increased requirement and transport of Trp to the fetus (Table 4). Thus, umbilical cord total and free [Trp] are in most cases 70%–108% and 68%–125% respectively higher than in maternal plasma. Plasma free and total [Trp] are also high at birth, but quickly decline within 24 h and reach normal values by day 7 of life [30].

**Table 4:** Trp concentrations in maternal, umbilical cord and newborn plasma

Reference	Maternal		Cord		Newborn	
	Total Trp	Free Trp	Total Trp	Free Trp	Total Trp	Free Trp
[30]	53	12.2	101	20.5	58	7.0
[31]	46	13.7	*	*	91	22.7
[33]	33	6.4	56	10.9	*	*
[34]	40	7.5	83	16.9	*	*
[35]	35	6.3	72	7.0	*	*
[51]	*	*	*	*	93	18.7

*Not determined.

For obvious reasons, little work has been done to assess human placental and fetal Trp disposition. In one study, in pregnancy terminated for psychological reasons [52], fetal plasma total [Trp] at 12–17 weeks of gestation was 1.5-fold that of maternal [Trp]. More fetal studies have been performed in experimental animals. In a control group of female rats in an alcohol-related study [53], [Trp] in maternal plasma and liver, placenta and fetal plasma, liver and brain were 51, 73, 105, 132, 144 and 104 μM respectively. In mice, [Trp] in maternal plasma and liver, placenta and whole fetuses were 170, 80, 110 and 200 μM respectively [54]. These data demonstrate the increased requirement of the fetus for Trp. In addition to its essential role in protein synthesis and hence fetal growth, Trp requirements extend to the importance its metabolites have in pregnancy.

### Serotonin for fetal and newborn development

One such metabolite is 5-HT, whose synthesis involves hydroxylation of Trp by Trp hydroxylase (TPH) followed by decarboxylation of 5-hydroxytryptophan by aromatic L-amino acid decarboxylase. Hydroxylation is the rate-limiting step. Most of the body 5-HT is synthesized in the periphery by the TPH1 isoform, whereas TPH2 participates in neuronal 5-HT synthesis. Because TPH is unsaturated with its Trp substrate, cerebral 5-HT synthesis is limited by circulating Trp availability to the brain, which is controlled primarily by liver TDO activity and secondarily by extent of competition for entry into the brain, mainly from five competing amino acids (CAA), namely valine, leucine, isoleucine, phenylalanine and tyrosine and Trp binding to albumin [5,7]. Trp availability to the brain is therefore expressed as the free or total [Trp]/[CAA] ratio. The increased maternal plasma free or/and total [Trp] throughout pregnancy ensures that Trp availability to the brain and hence cerebral 5-HT synthesis will not be impaired. This is important, as the behavioural consequences of a 5-HT dysfunction in mother can reflect negatively on newborn developmental health. Despite the decrease in total [Trp] in late pregnancy, the total [Trp]/[CAA] ratio is either normal [37] or slightly decreased [38], because [CAA] are also decreased, presumably as a result of increased placental extraction of amino acids [55]. As free [Trp] is elevated in pregnancy, the free [Trp]/[CAA] ratio can be assumed to be increased. The absence of a 5-HT deficiency during pregnancy is consistent with the absence of depression, as most women who are depressed during pregnancy are also depressed in the months preceding and following pregnancy ([56] and references cited therein). It is of interest in this context that oestrogens and progesterone exert anti-depressant effects in postnatal depression and that oestrogens accelerate the anti-depressant effects of specific 5-HT -reuptake inhibitors (see [7] for references).

An adequate supply of 5-HT is vital for fetal development [57,58] and myometrium contractions [59]. The 5-HT sources are the maternal circulation in early and mid-pregnancy [60] and the placenta thereafter [61].

### Kynurenine pathway enzymes and metabolite formation in pregnancy

Production of K metabolites is achieved by the flux of Trp down the KP, without the need for induction of any particular enzyme, as long as the enzymes are functionally expressed. It is therefore important to discuss first the status of enzymes of the pathway in pregnant females, placental and fetal tissues.

All enzymes of the pathway are present in maternal liver, whereas only a limited number exist in extrahepatic tissues. From the above discussion, liver TDO is inhibited in early and mid-pregnancy and there is no evidence for its induction at any stage of pregnancy. Extrahepatic IDO activity (outside the placenta) has not been studied in human pregnancy and the notion that it may be induced in late pregnancy on the basis of an increase in the maternal plasma [K]/[Trp] ratio [36,39] is not supported, because of the fortuitous nature of this increase [48], being due to the decrease in [Trp] (through increased tissue uptake) without a concomitant increase in [K] or information on free [Trp]. The use of an increase in the [K]/[total Trp] ratio as an expression of IDO/TDO induction is of course valid under conditions wherein such induction is both assured and robust, e.g. with glucocorticoids (TDO) or IFN-γ under many pathological conditions (IDO).

TDO, IDO and subsequent enzymes of the KP are expressed in human and mouse placentas and concepti [54,62–67]. Of relevant interest are the findings that, in addition to IDO induction, cytokines released in response to infection with bacterial endotoxin (lipopolysaccharide) enhance the activities of placental TDO, kynureninase and 3-HAA oxidase [66,67] and that progesterone inhibits IDO mRNA expression and enzyme activity during decidualization of the endometrium [68]. In mice [69], IDO protein and mRNA in concepti are expressed only in mid-pregnancy (day 8.5), lasting only for 3 days before declining rapidly. IDO appearance coincides with placental formation. By contrast, TDO expression occurs earlier (day 5.5), is maintained for 7 days and is still detectable on day 18.5 [69]. Most of the Trp degrading activity in concepti up until placental formation on day 10.5 is attributable to TDO, whose highest activity is >3-fold that of the adult hepatic enzyme. By the time IDO is expressed on days 8.5–12.5, the Trp-degrading activity has already receded to low levels before ceasing completely on day 14.5. Even when the IDO mRNA is maximally expressed on day 9.5, the Trp-degrading activity of concepti is not impaired by the IDO inhibitor 1-MT [69]. Taken together, this evidence suggests that: (1) Trp degradation by concepti in early to mid-pregnancy is catalysed by TDO; (2) the role of IDO in mid-pregnancy is both partial and transient; (3) Trp degradation in mid-pregnancy may therefore be achieved by both IDO and TDO. In humans, urinary excretion of Trp and niacin metabolites is enhanced in mid- and late pregnancy [70], suggesting that Trp degradation down the KP is most active during these two trimesters. It is not clear where or which enzyme(s) contributes to K metabolite formation in late pregnancy. Studies of the role of IDO in this regard are controversial, possibly due to methodological differences ([64] and references cited therein) and it has been suggested that a shift from syncytiotrophoblast to fetal endothelial cells in terminal villi indicates that IDO may perform other functions in late gestation [65]. The above changes also suggest that, as the Trp-degrading activity is maximal at mid- and late pregnancy, this may be the time at which immune tolerance is achieved through the immunosuppressant effects of K metabolites (see below).

### Kynurenic acid for neuronal protection

The K transamination product KA is the only known physiological antagonist of the *N*-methyl-D-aspartate (NMDA) receptors of the excitatory amino acid glutamate [71]. Its main function is neuronal protection against hyperexcitability and anxiety and an increase in its availability to the fetus is therefore desirable. As stated earlier, transamination of K to KA is normally a minor reaction, but can be enhanced after loading with Trp or K. Cerebral KA synthesis is best enhanced when plasma [K] is elevated, but is only moderately increased by Trp loading [72], thus reflecting the low cerebral IDO activity. [KA] in umbilical cord blood (38.9 nM) is nearly twice that (20.5 nM) in maternal blood [34], reflecting the almost 3-fold higher [K] in cord blood [34]. Urinary KA excretion is enhanced in pregnant rats, though not in pregnant women [70]. KA levels in fetal tissues cannot be predicted from these increases, but since the latter occur in normal pregnancy, it must be assumed that undesirably high levels do not occur to undermine brain function.

### Other Ks for immunosuppression

There are many reasons for rejecting the earlier concept [3] that starving bacteria, parasites and viruses of Trp undermines their infectivity. These include the ability of bacteria to synthesize Trp in the presence of Trp depletion [73] and of the malaria parasite to survive and grow in mice, despite IDO induction and the consequent Trp depletion [74] and the absence of a correlation between Trp depletion within human ovarian tumour xenographs in mice and the anti-tumour response to IFN-γ [75]. A detailed account of evidence against the Trp starvation concept led Moffett and Namboodiri [76] to propose a Trp utilization concept as a comprehensive theory of immune-related IDO induction, with particular emphasis on the role of QA in NAD^+^ synthesis and the tissue distribution of IDO and TDO in health and infectious disease.

Part of this evidence relates to the ability of some K metabolites to suppress T-cell responses. Thus, 3-HK and 3-HAA suppress allogeneic T-cell proliferation in an additive manner, most probably by an apoptotic mechanism [77]. This mechanism has been suggested to explain the ability of 3-HAA and QA to undermine T helper type 1 (Th1) cells [78]. Inhibition of 3-HK synthesis by a K hydroxylase inhibitor (Ro-61-8048) results in prolonged survival of a murine model of cerebral malaria [79], presumably not involving Trp depletion. The mechanism of immune suppression by K metabolites is thought to involve selective apoptosis of Th1 cells [78], thus shifting the Th1/Th2 balance towards Th2 reactivity.

### Vital role of tryptophan flux in kynurenine metabolite formation in pregnancy

In the above two studies [77,78], the concentrations of 3-HK and 3-HAA required for suppression are relatively high (10 μM) or higher. It is not clear if these concentrations could be reached *in vivo* in localized compartments even if IDO or TDO is induced in the absence of an increase in Trp availability and hence flux down the KP, which can occur either through exogenous Trp loading or by endogenous mechanisms. For example, single oral Trp doses of 5.15–10.30 g (equivalent to 74–147 mg/kg for a 70 kg adult), which can also activate TDO, are necessary to elevate human plasma [K] and [3-HK] to values above 10 μM (A.A.-B. Badawy and D.M. Dougherty, unpublished work). Elevation of 3-HAA is less striking, possibly because of its rapid oxidation and conversion to QA (see below). In fact, the maximum increase in rat liver [3-HAA] occurs at 1 h after its intraperitoneal administration, with a 5 mg/kg body weight dose causing the maximum increase [80]. Because K undergoes a significant renal clearance [81,82], its plasma levels can only be increased by inhibition of K hydroxylase and by K or Trp loading or TDO/IDO induction. For example, IDO induction in seropositive HIV patients produces a 49% increase in serum [K] (from 2.31 to 3.45 μM) and a 37% decrease in [Trp] from 91 to 57 μM [83]. Reversing these percentages to ratios reveals a reasonable concordance (0.0204 for the K and 0.0213 for the Trp, changes). By contrast, increased flux of Trp (without TDO induction) can increase K metabolite formation more dramatically. Thus, a 50 mg/kg oral Trp dose (∼3.5 g for a 70 kg adult) increases plasma [K] in healthy female subjects from 2.5 to 22 μM [84]. Intravenous Trp doses of 1 and 3 g increase plasma [K] in male subjects from to 3 μM at baseline to 12 and 22 μM respectively at 4 h [85]. Also, flux through TDO has been shown to be enhanced in isolated hepatocytes from leucine-fed rats [86] and in human volunteers given leucine with 15 other amino acids including Trp in a control formulation for the acute Trp depletion test [87]. In the absence of added Trp (i.e. with the Trp depletion formulation), leucine enhances the flux of Trp down the pathway, increasing the plasma concentrations of 3-HK and 3-HAA by 12.8- and 2.85-fold respectively, despite decreases in free Trp, total Trp and K of 85%, 81% and 45% respectively (A.A.-B. Badawy and D.M. Dougherty, unpublished work). Also, the liver of a rat in the absence of TDO induction can metabolize the whole of its Trp every 7 min or faster [88]. Taken together, this suggests that, for significant amounts of immunosuppressive Ks to be formed, the flux of Trp down the pathway must be enhanced by an increase in Trp availability and irrespective of whether TDO or IDO is induced. The primary requirements in pregnancy are therefore elevation of plasma free Trp availability and expression of Trp-degrading enzymes in conceptus, placenta and fetus.

Little is known about levels of immunosuppressive and other Ks in pregnancy. Compared with maternal blood, fetal umbilical cord blood in normal pregnancy exhibits elevated levels of K (2.5–5.2-fold: 4.3–7.9 μM) and 3-HAA (6.6–58.3-fold: 0.27–0.70 μM). Umbilical cord blood contains QA (1.6 μM) and picolinic acid (1.7 μM), but lower levels of KA (0.04–0.42 μM) and xanthurenic acid (0.19 μM) [34,35,66]. These values for K, 3-HAA, KA and QA do not approach those encountered in pathological conditions and are mostly similar to or moderately higher than normal plasma values in the literature.

### Quinolinic acid for NAD^+^ synthesis

Although QA is excitotoxic, it is nevertheless the obligatory precursor of the all-important cofactor NAD^+^, necessary for a wide array of redox reactions and DNA repair and its phosphorylated form NADP^+^, involved in many biosynthetic redox reactions. Trp, but not niacin, is the more effective source of NAD^+^(P^+^) synthesis [89] and this may be facilitated by the relatively very high activity of hepatic 3-HAA oxidase [90] ensuring high production of QA. This notion is supported by the finding that relatively large amounts of QA are found in urine of pregnant women [70]. The 44–58-fold increase in [3HAA] in umbilical cord blood over that in maternal blood [34] strongly suggests that adequate amounts of QA are available for fetal NAD^+^ synthesis. As stated above, QA also possesses immunosuppressive properties [78].

## TRYPTOPHAN AND ABNORMAL PREGNANCY

Trp metabolism may also play an important role in abnormal pregnancies. A potential role of excessive levels of Trp in pre-eclampsia (PE) is suggested from a variety of findings, most importantly the observation [91] that high Trp levels can undermine T-cell suppression by K metabolites. In mice fed a high (5%) Trp diet, decreases in placental, fetal body and pup weights and increased mortality of pups were observed [54]. It can thus be assumed that levels of Trp are regulated during normal pregnancy to establish a balance between the physiological needs of the fetus and safeguarding it against rejection. Consequently, loss of this control can jeopardise pregnancy in two ways: (1) severe malnutrition can cause a strong Trp deficiency and hence decreased production of immunosuppressing Ks, thus depriving the fetus from this essential nutrient and undermining suppression of T-cell responses; (2) an excessive increase in Trp is likely to reverse the immunosuppression by Ks, resulting in pregnancy complications. An excess of Trp could occur in PE because of increased placental amino acid transport [92] (though Trp was not measured in the latter study) or if Trp release from albumin-binding sites is enhanced through changes in both [albumin] and [NEFA] beyond those observed in normal pregnancy, leading to increased flux down the KP. Increased flux is suggested by the observed increase in QA in PE [93] and toxaemia of pregnancy [94].

### Possible role of albumin

In a study of pregnant women of comparable age and pregnancy durations (35–37 weeks), serum [albumin] was shown [95] to be decreased, relative to non-pregnant controls, by 31% in normal pregnancy, 37% in PE without proteinuria and 50% in PE with proteinuria. Values below 20 g/l have been associated with severe PE, though not as an independent variable [96]. Studies in isolated rat hepatocytes [9] with albumin added to various concentrations of Trp showed that at an albumin level of 20 g/l and a [Trp] of 100 μM (some 67% higher than that in human plasma), free [Trp] is 27% of the total. We could therefore expect an-even higher value, at least 30%, in PE.

### Possible role of NEFA

By contrast, [NEFA] are excessively elevated in PE [45,97–99] over the maximum 44% elevation in the third trimester of normal pregnancy [46], with (additional) increases compared with normal pregnancy of 68% [97], 78% [98], 97% [99] and 393% [45]. Thus, compared with non-pregnant controls (100%), the [NEFA] values in PE are 235%–690%, compared with 144% in normal pregnancy. These huge elevations are almost certain to induce a strong release of albumin-bound Trp into the maternal circulation. Spontaneous pre-term delivery is also associated with greater [NEFA] than normal-term delivery [44,100]. Trp binds to albumin at a single high-affinity binding site with an association constant of 4.4×10^−4^ M, a 1:1 molar ratio and in competition with NEFA [101]. By contrast, NEFA can occupy several binding sites, the primary site being different from the Trp-binding site [102]. A molar NEFA/albumin ratio of 1 does not alter the affinity or number of binding sites for Trp, whereas at a ratio of two or above, the number of Trp-binding sites is gradually decreased [102]. Thus extent of Trp displacement is a function of [NEFA]. At a [NEFA] of 2 mEq/l, 50% of plasma Trp exists in the free state [103]. NEFA can also aggravate the immune state by precipitating oxidative stress leading to a pro-inflammatory environment [104–106].

### Combined effects of albumin and NEFA

When [NEFA] are strongly elevated in the presence of a sizeable decrease in [albumin], as in PE, we can expect a large increase in free [Trp]. This important parameter has not been measured in PE, but can be predicted from data obtained in other conditions associated with the above changes in NEFA and albumin, namely liver diseases. For example, plasma free [Trp] is elevated from a 4 μM in control subjects to 21 μM in patients with fulminant hepatic failure [107] (an increase in the percentage free Trp from 7.7% to 41.2% or a 435% increase). In another study [108], free [Trp] was elevated from 11 μM in control subjects to 56 μM in patients with liver cirrhosis with oesophageal varices and to 107 μM in patients with fulminant hepatitis. Although plasma total [Trp] is progressively elevated in these hepatic conditions, because of impaired degradation by TDO, the percentage free was still elevated from 22% in controls to 55% and 95% respectively (reflecting increases of 150%–332%). These huge elevations can be contrasted with the 58%–100% increases in the percentage free Trp in normal human pregnancy (Table 3) and the 180% increase in rat pregnancy [6].

### How much tryptophan is required to undermine immune suppression by kynurenines during pregnancy?

The mechanism(s) by which an excess of Trp harms pregnancy is not currently understood. A daily Trp supplement of up to 5 g does not exert a negative effect on human pregnancy [109]. A single 5.15 g oral Trp dose has been shown [110] to maximally increase plasma free and total [Trp] in healthy volunteers of both genders from the zero-time values of 4.7 and 56 μM respectively to 76 and 491 μM respectively at 3 h. Smaller increases can therefore be expected with divided doses. For example, the corresponding values for a 1.15 g dose are 4.8 and 53 μM at zero-time and 11.3 and 98 μM at 3 h [111]. The precise [Trp] required to undermine T-cell suppression by Ks is currently a matter for speculation. In a study with monocytes [91], the [Trp] in the medium was 490 μM, a concentration similar to that used in a study [112] demonstrating the ability of Trp to block the anti-microbial effects of recombinant TDO protein expressed in HeLa cells. It is noteworthy that the [Trp] required to maintain bacterial growth is some 40-fold higher than that needed by monocytes [113]. Also, protein binding in medium (possibly trapping 90%) and restricted cellular uptake, possibly by competition from other amino acids in the culture medium, suggest that a much smaller concentration enters the cells. These considerations and the known increase in Trp transport suggest that the excessive increase in free [Trp] in PE may be sufficient to undermine the immunosuppressant effects of Ks.

## IMMUNE STATUS IN PREGNANCY IN RELATION TO TRYPTOPHAN METABOLISM

Whereas a discussion of the extensive literature of the immune status in pregnancy is outside the scope of the present review, it is important to attempt briefly to follow the time-course of changes in the immune status in parallel with those in Trp metabolism discussed above to establish the role or response of the latter to changes in the former. Most immune studies in pregnancy have been performed in the third trimester, with very few in the first, largely because some women see no need to comply with appointments at this stage. Results of these studies have in general been contradictory to some extent, because of small sample sizes, absence of a non-pregnant control group or use of analytical methodologies of varying sensitivities. Despite these limitations, the following conclusions can be drawn from the longitudinal and other studies described below.

### First trimester

A multiplex array study of maternal cytokines across the entire time-course of pregnancy in 16 subjects [114] found that 50% of the 42 cytokines measured did not show an association with gestational weeks or trimester. The first trimester is characterized by an increase in growth factors and a relative decrease in inflammatory markers, despite the predominance of Th1 cells (5%–30%) relative to Th2 (5%) and Th17 (2%) among decidual CD4+ cells. In another (bioplex) study of 44 subjects in the first trimester [115], pro-inflammatory cytokines were undetectable and of no predictive value for miscarriage. An increase in the anti-inflammatory cytokines interleukin-35 and transforming growth factor-beta (IL-35 and TGF-β) has been reported in the first trimester in 120 subjects [116] and it was suggested that IL-35 plays an important role in normal pregnancy, as a decrease in its level is associated with recurrent spontaneous abortion. These findings strongly suggest that a significant pro-inflammatory environment does not exist in mother in early pregnancy and that, consequently, immunosuppression by K metabolites is not required at this stage. The potential inhibition of human maternal liver TDO activity by the elevated levels of oestrogens and progesterone, as in rats [6], is consistent with the notion that emphasis in early pregnancy is on provision of Trp for protein and 5-HT synthesis in mother and conceptus and NAD^+^ synthesis from QA in conceptus through TDO, but not IDO, expression, as shown in mice [70].

In humans however, evidence exists for a functionally expressed IDO in first trimester decidua [65] and it is therefore likely that its function at this stage may be limited primarily to catalysis of Trp oxidation, rather than immune related activities, given the absence of a pro-inflammatory environment at this stage of pregnancy.

### Second and third trimesters

Results of studies during these two trimesters are controversial with some demonstrating increased pro-inflammatory cytokine levels (IL-1β, IL-6, IL-12, IL-15, interferon-gamma-induced protein 10 (IP-10) and soluble CD40 ligand (sCD40) (CD40) ligand and IFN-γ) [117,118] and others showing decreases [119,120]. Heterogeneity and sample size differences may explain this controversy. Another potential explanation is that of possible differences in levels of immunosuppressive K metabolites and/or their Trp precursor, measurements of which may be informative. These differences apart, a pro-inflammatory environment is likely to occur in mid- to late pregnancy, the source of which may not be the mother (see below). The second trimester coincides with placental formation. Immunosuppression may be required at this stage, at which time TDO, IDO and other enzymes of the KP are expressed. Little is known regarding maternal and/or or umbilical cord blood K metabolite levels at the different stages of pregnancy. Only the increases described above [34,35,66], just before or at parturition, have been reported. At parturition, amniotic fluid pro-inflammatory (IL-1β, IL-6, IL-8, tumour necrosis factor-alpha (TNF-α) and IFN-γ) and anti-inflammatory (IL-10 and interlreukin-1 receptor antagonist (IL-1ra)) cytokine levels are higher than in cord blood or maternal plasma [121]. The preponderance of pro-inflammatory cytokines coincides with the onset of labour. Gender differences could be seen from the observed [121] higher levels of pro-inflammatory cytokine in amniotic fluid in male than in female fetuses and the particularly higher IL-1ra in the latter fetuses. The protective effect of this latter cytokine may explain the lower incidence of pre-term births, still births and unexpected deaths in infancy among females.

### Abnormal pregnancy

The relationship between the maternal immune response during pregnancy and the onset of PE has recently been reviewed [122]. More clear-cut changes in cytokine levels have been reported in abnormal pregnancy. Thus, maternal levels of IL-1, IL-6, IL-8, TNF-α, IFN-γ, soluble interleukin-4 receptor (sIL-4r) and C-reactive protein are elevated in PE, preterm births or intrauterine growth retardation [123–130]. A potential excessive elevation of free [Trp] in abnormal pregnancies could result in a simultaneous increase in immunosuppressive Ks. Plasma free [Trp] has indeed been shown to be elevated in newborn babies with intrauterine growth restriction [131]. The excess Trp can still undermine this suppression despite the enhanced elevation of Ks. Until Trp and K metabolite levels are studied simultaneously in abnormal pregnancy, this possibility remains a matter of speculation. In a large population study [132] of Trp and its K metabolites, only elevated KA in maternal plasma in early pregnancy was associated with increased risk of PE in obese women. However, non-fasting plasma samples were used, which could have confounded the study outcome. It is noteworthy that Trp metabolism, including changes in kynureninase and aminomuconic semialdehyde dehydrogenase, features highly in a canonical pathway analysis in a genome-wide study in PE [133].

## GENERAL CONCLUSIONS AND FUTURE PERSPECTIVES

The role of Trp in pregnancy is better expressed in terms of a utilization concept. The pregnant mother provides: (1) the precursors necessary for fetal growth and development (nutrients, including Trp and developmental mediators) and (2) the means to protect the fetus against rejection by immunosuppressive K metabolites, but also (3) has the ability to reject in an emergency.

In relation to protecting the fetus, the primary event may be the increase in Trp availability, suggested by the greater content of Trp in umbilical cord blood than in maternal circulation. Trp depletion can therefore be excluded as the protective mechanism and evidence suggests that protection may be achieved by immunosuppressive K metabolites. The flux of Trp down the KP is vital for production of these K metabolites, whose cord blood levels are elevated, as long as the necessary enzymes are functionally expressed in placenta and fetal tissues. There is no evidence that maternal TDO or IDO is enhanced in pregnancy and any likely enhancement in placenta does not deprive the fetus of Trp. The finding [15] that pregnancy can proceed to a successful conclusion regarding fetal survival after IDO1 gene deletion further suggests that IDO does not play a primary role in pregnancy. It is of interest that IDO1 gene-deletion resulted in a PE phenotype characterized, among others, by proteinuria [15]. Under these conditions, plasma albumin could be decreased by as much as 50% (see above), resulting in excessive elevation of free [Trp], as suggested earlier. In an emergency, immunosuppression is undermined by a mechanism(s) as yet unclear. Of potential interest is the excessive elevation of NEFA leading to oxidative stress and a consequently pro-inflammatory environment. Trp availability is likely to be further increased under these conditions and pregnancy outcome may depend on the relative increases in Trp and K metabolites. If TDO or IDO is fully substrate-saturated, the excess Trp may undermine the immune suppression. The possibility that the fetus itself elicits a maternal rejection response is supported in a study of a series of dizygotic twins demonstrating an association between severity of chronic villitis and small birth weight [134].

Many important questions need to be addressed in future studies. These include: (1) measuring levels of free Trp in conjunction with those of total Trp, albumin, NEFA, K and its metabolites and immune parameters in maternal plasma (and where appropriate placenta, cord blood and amniotic fluid) in/throughout normal and abnormal pregnancies; (2) assessing the role of K metabolites as immunosuppressants as a function of [Trp] in suitable cell systems (3) assessing the potential effect of excess Trp on pregnancy outcome in experimental models in conjunction with immune parameters. If changes in Trp disposition are shown to play a role in abnormal pregnancies, suitable interventions could be considered. For example, albumin infusions have been used in PE to correct for the albumin depletion and, although this intervention does not ameliorate the hypertension or the decreased intervillous blood flow, it may restrict the flux of Trp if this is deemed necessary. It is hoped that such studies can provide an insight into the dynamics of pregnancy and help identify treatment strategies aimed at ensuring a safe outcome.

## Abbreviations

3-HAA: 3-hydroxyanthranilic acid
3-HK: 3-hydroxykynurenine
5-HT: 5-hydroxytryptamine or serotonin
CAA: competing amino acids
IDO: indoleamine 2,3-dioxygenase
IFN-γ: interferon-γ
IP-10: interferon-gamma-induced protein 10
IL-: interleukin
IL-1ra: interleukin-1 receptor antagonist
K: kynurenine
KA: kynurenic acid
KP: kynurenine pathway
LPS: lipopolysaccharide (endotoxin)
1 MT: 1 methyltryptophan
NAD+(P+)H: oxidized and reduced nicotinamide-adenine dinucleotide (phosphate)
NEFA: non-esterified fatty acids
NMDA: *N*-methyl-D-aspartate
PE: pre-eclampsia
QA: quinolinic acid
TDO: tryptophan 2,3-dioxygenase
Th1: T helper type 1
TGF-beta: transforming growth factor-beta
TPH: Trp hydroxylase
Trp: tryptophan
TNF-alpha: tumour-necrosis factor-alpha
XA: xanthurenic acid

## References

[1] Munn D.H., Zhou M., Attwood A.J., Bondarev I., Conway S.J., Marshall B., Brown C., Mellor A.L. (1998). Prevention of allogeneic fetal rejection by tryptophan catabolism. Science.

[2] Pfefferkorn E.R., Rebhun S., Eckel M. (1986). Characterisation of an indoleamine dioxygenase induced by gamma interferon in cultured human fibroblasts. J. Interferon Res..

[3] Taylor M.W., Feng G.S. (1991). Relationship between interferon-gamma, indoleamine 2,3-dioxygenase, and tryptophan catabolism. FASEB J..

[4] Bender D.A. (1983). Biochemistry of tryptophan in health and disease. Mol. Aspects Med..

[5] Badawy A.A.-B. (2002). Tryptophan metabolism in alcoholism. Nutr. Res. Rev..

[6] Badawy A.A.-B. (1988). Effects of pregnancy on tryptophan metabolism and disposition in the rat. Biochem. J..

[7] Badawy A.A.-B. (2013). Tryptophan: the key to boosting brain serotonin synthesis in depressive illness. J. Psychopharmacol..

[8] Yamazaki F., Kuroiwa T., Takikawa O., Kido R. (1985). Human indolylamine 2,3-dioxygenase, its tissue distribution, and characterization of the placental enzyme. Biochem. J..

[9] Smith S.A., Pogson C.I. (1980). The metabolism of *L*-tryptophan by isolated rat liver cells: effect of albumin binding and amino acid competition on oxidation of tryptophan by tryptophan 2,3-dioxygenase. Biochem. J..

[10] Badawy A.A.-B., Evans M. (1976). Animal liver tryptophan pyrrolases–absence of apoenzyme and of hormonal induction mechanism from species sensitive to tryptophan toxicity. Biochem. J..

[11] Hucke C., Mackenzie C.R., Adjogble K.D.Z., Takikawa O., Däubener W. (2004). Nitric oxide-mediated regulation of gamma interferon-induced bacteriostasis: inhibition and degradation of human indoleamine 2,3-dioxygenase. Infect. Immun..

[12] Kröncke K.-D., Fehsel K., Kolb-Bachefen V. (1998). Inducible nitric oxide synthase in human tissues. Clin. Exp. Immunol..

[13] Allegri G., Bertazzo A., Biasiolo M., Costa C.V., Ragazzi E. (2003). Kynurenine pathway enzymes in different species of animals. Adv. Exp. Med. Biol..

[14] Kanai M., Funakoshi H., Takahashi H., Hayakawa T., Mizuno S., Matsumoto K., Nakamura T. (2009). Tryptophan 2,3-dioxygenase is a key modulator of physiological neurogenesis and anxiety-related behaviour in mice. Mol. Brain.

[15] Santillan M.K., Pelham C.J., Gibson-Corley K.N., Scroggins S.M. (2015). Pregnant mice lacking indoleamine 2,3-dioxygenase exhibit preeclampsia phenotypes. Physiol. Rep..

[16] Nakamura T., Niimi S., Nawa K., Noda C., Ichihara A., Takagit Y., Anaiq M., Sakaki Y. (1987). Multihormonal regulation of transcription of the tryptophan 2,3-dioxygenase gene in primary cultures of **a**dult rat hepatocytes with special reference to the presence of a transcriptional protein mediating the action of glucocorticoids. J. Biol. Chem..

[17] Liao M., Pabarcus M.K., Wang Y., Hefner C., Maltby D.A., Medzihradszky K.F., Salas-Castillo S.P., Yan J., Maher J.J., Correia M.A. (2007). Impaired dexamethasone-mediated induction of tryptophan 2,3-dioxygenase in heme-deficient rat hepatocytes: translational control by a hepatic eIF2_kinase, the heme-regulated inhibitor. J. Pharmacol. Exp. Ther..

[18] Cook J.C., Pogson C.I., Smith S.A. (1980). Indoleamine 2,3-dioxygenase: a new, rapid sensitive radiometric assay and its application to the study of the enzyme in rat tissues. Biochem. J..

[19] Badawy A.A.-B. (2010). Perspective: plasma free tryptophan revisited: what you need to know and do before measuring it. J. Psychopharmacol..

[20] Monroe C.N.B. (1968). Induction of tryptophan oxygenase and tyrosine aminotransferase in mice. Am. J. Physiol..

[21] Badawy A.A.-B., Evans M. (1976). The role of free serum tryptophan in the biphasic effect of acute ethanol administration on the concentrations of rat brain tryptophan, 5-hydroxytryptamine and 5-hydroxyindol-3-ylacetic acid. Biochem. J..

[22] Badawy A.A.-B., Morgan C.J., Lane J., Dhaliwal K., Bradley D.M. (1989). liver tryptophan pyrrolase: a major determinant of the lower brain 5-hydroxytryptamine concentration in alcohol-preferring C57BL mice. Biochem. J..

[23] Bano S. (1997). Tryptophan Metabolism in Relation to Mental Illness.

[24] Soudi S., Zavaran-Hosseini A., Hassan M.Z., Soleiman M., Adegani J.F., Hashemi S.M. (2013). Comparative study of the effects of LPS on the function of BALB/c and C57BL/6 peritoneal macrophages. Cell J..

[25] Carrera-Silva E.A., Cano R.C., Guiñazu N., Aoki M.P., Pellegrini A., Gea S. (2008). TLR2, TLR4 and TLR9 are differentially modulated in liver lethally injured from BALB/c and C57BL/6 mice during *Trypanosoma cruzi* acute infection. Mol. Immunol..

[26] O'Conner M.A, Green W.R. (2013). The role of indoleamine 2,3-dioxygenase in LP-BPMS5 murine retroviral disease progression. Virol. J..

[27] Kudo Y., Boyd C.A.R. (2001). The role of *L*-tryptophan transport in *L*-tryptophan degradation by indoleamine 2,3-dioxygenase in human placental explants. J. Physiol..

[28] Vumma R., Johansson J., Lewander T., Venizelos N. (2011). Tryptophan transport in human fibroblast cells–a functional characterization. Int. J. Tryptophan Res..

[29] Opitz C A., Litzenburger U.M., Opitz U., Sahm F., Ochs K., Lutz C., Wick W., Platten M. (2011). The indoleamine-2,3-dioxygenase (IDO) inhibitor 1-methyl-D-tryptophan upregulates IDO1 in human cancer cells. PLoS One.

[30] Tricklebank M.D., Pickard F.J., de Souza S.W. (1979). Free and bound tryptophan in human plasma during the perinatal period. Acta Paediatr. Scand..

[31] De Antoni A., Carretti N., Costa C., D'Antona N., Bertolin A., Allegri G. (1980). Triptofano totale e libero nel siero di madri al momento del parto e dei rispettivi neonati (serum total and free tryptophan concentration in mothers at delivery and in their newborn babies). Giorn. It. Chim. Clin..

[32] Handley S.L., Dunn T.L., Waldron G., Baker J.M. (1980). Tryptophan, cortisol and puerperal mood. Br. J. Psychiat..

[33] Zanardo V., Casson L., Costa C., Biasiolo M., Allegri G., Vicenti E., Grella P. (1985). Level of total and free tryptophan in maternal and in umbilical blood of infants born by vaginal delivery and by elective cesarian section. Biol. Neonate.

[34] Kamimura S., Katsuto E., Sekiba K. (1991). Tryptophan and its metabolite concentrations in human plasma and breast milk during the perinatal period. Acta Med. Okayama.

[35] Morita I., Kawamoto M., Yoshida H. (1992). Difference in the concentration of tryptophan metabolites between maternal and umbilical foetal blood. J. Chromatogr..

[36] Schröcksnadel H., Baier-Bitterlich G., Dapunt O., Wachter H., Fuchs D. (1996). Decreased plasma tryptophan in pregnancy. Obstet. Gynecol..

[37] Abou-Saleh M.T., Ghubash R., Karim L., Krymski M., Ibrahim A. (1998). Postpartum mood changes and plasma amino acids. Curr. Psychiat..

[38] Maes M., Ombelet W., Verkerk R., Bosmans E., Scharpé S. (2001). Effects of pregnancy and delivery on the availability of plasma tryptophan to the brain: relationships to delivery-induced immune activation and early post-partum anxiety and depression. Psychol. Med..

[39] Kudo Y. (2013). The role of placental indoleamine 2,3-dioxygenase in human pregnancy. Obstet. Gynecol. Sci..

[40] Bloxam D.L., Warren W.H. (1974). Error in the determination of tryptophan by the method of Denckla and Dewey. A revised procedure. Anal. Biochem..

[41] Sherlock S., Sherlock S. (1981). The liver in pregnancy.

[42] Abbassi-Ghanavati M., Greer L.G., Cunningham G.F. (2009). Pregnancy and laboratory studies: a reference table for clinicians. Obstet. Gynecol..

[43] Laron Z., Mannheimer S., Nitzan M., Goldmann J. (1967). Growth hormone, glucose and free fatty acid levels in mother and infant of normal, diabetic and toxaemic pregnancies. Arch. Dis. Child..

[44] Chen X., Scholl T.O. (2008). Association of elevated free fatty acids during late pregnancy with preterm delivery. Obstet. Gynecol..

[45] El Beltagy N.S., Sadek S.S. E., Zidan M.A., Abd El Naby R.E. (2011). Can serum fatty acids assessment predict severe preeclampsia?. Alexandria J. Med.

[46] Fairweather D.V.I. (1971). Changes in levels of serum non-esterified fatty acid and blood glucose in pregnancy. Br. J. Obstet, Gynaecol. Br. Commonw..

[47] Altman K., Greengard O. (1966). Correlation of kynurenine excretion with liver tryptophan pyrrolase levels in disease and after hydrocortisone induction. J. Clin. Invest..

[48] Badawy A.A.-B. (2014). Review: the tryptophan utilization concept in pregnancy. Obstet. Gynecol. Sci..

[49] World Health Organization (2007). Protein and amino acid requirements in human nutrition.

[50] Moe A.J. (1995). Placental amino acid transport. Am. J. Physiol..

[51] De Antoni A., Allegri G., Costa C., Vanzan S., Bertolin A., Carretti N., Zanardo V. (1980). Total and free tryptophan levels in serum of newborn infants. relationship with the serotonin and nicotinic acid pathways. Acta Vitaminol. Enzymol..

[52] Jauniaux E., Gulbis B., Gerloo E. (1999). Free amino acids in human fetal liver and fluids at 12-17 weeks of gestation. Hum. Reprod..

[53] Lin G.W.-J., Lin T.-Y., Jin L. (1990). Gestational ethanol consumption on tissue amino acid levels: decreased free histidine and tryptophan in fetal tissue with concomitant increase in urinary histamine excretion. Alcohol. Clin. Exp. Res..

[54] Tsuji A., Nakata C., Sano M., Fukuwatari T., Shibata K. (2013). L-tryptophan metabolism in pregnant mice fed a high L-tryptophan diet and the effect on maternal, placental and fetal growth. Int. J. Tryptophan Res..

[55] Camelo J.S., Martinez F.E., Gonçalves A.L., Monteiro J.P., Jorge S.M. (2007). Plasma amino acids in pregnancy, placental intervillous space and preterm newborn infants. Braz. J. Med. Biol. Res..

[56] Doornboss B. (2009). Hormones, monoamines and peripartum affective disorders.

[57] Moiseiwitsch J.R.D. (2000). The role of serotonin and neurotransmitters during craniofacial development. Crit. Rev. Oral Biol. Med..

[58] Doherty L.F., Kwon H.E., Taylor H.S. (2011). Regulation of tryptophan 2,3-dioxygenase by HOXA10 enhances embryo viability through serotonin signaling. Am. J. Physiol. Endocrinol. Metab..

[59] Cordeaux Y., Pasupathy D., Bacon J., Charnock-Jones D.S., Smith G.C. (2009). Characterization of serotonin receptors in pregnant human myometrium. J. Pharmacol. Exp. Ther..

[60] Côte´ F., Fligny C., Bayard E., Launay J.-M., Gershon M.D., Mallet J., Vodjdani G. (2007). Maternal serotonin is crucial for murine embryonic development. Proc. Natl. Acad. Sci. U.S.A..

[61] Bonnin A., Levitt P. (2012). Placental source of 5-HT that tunes fetal brain development. Neuropsychopharmacology.

[62] Alberati-Giania D., Cesuraa A.M., Brogera C., Warren W.D., Röver S., Malherbea P. (1997). Cloning and functional expression of human kynurenine 3-monooxygenase. FEBS Lett..

[63] Milart P., Urbańsca E.M., Turski W.A., Paszkowskia T., Sikoria R. (2001). Kynurenine aminotransferase I activity in human placenta. Placenta.

[64] Kudo Y., Boyd C.A.R., Spyropoulou I., Redman C.W.G., Takikawa O., Katsuki T., Hara T., Ohama K., Sargent L. (2004). Indoleamine 2,3-dioxygenase: distribution and function in the developing human placenta. J. Reprod. Immunol..

[65] Ligam P., Mannuelpillai U., Wallace E.M., Walker S. (2005). Localisation of indoleamine 2,3-dioxygenase and kynurenine hydroxylase in human placenta and decidua: implications for role of kynurenine pathway in pregnancy. Placenta.

[66] Mannuelpillai U., Ligam P., Smythe G., Wallace E.M., Hirst J., Walker D.W. (2005). Identification of kynurenine pathway enzyme mRNAs and metabolism in human placenta: upregulation by inflammatory stimuli and with clinical infection. Am. J. Obstet. Gynecol..

[67] Dharane Nee Ligam P, Mannuelpillai U., Wallace E.M., Walker D. (2010). NFkB-dependent increase of kynurenine pathway activity in human placenta: inhibition by sulfasalazine. Placenta.

[68] Kudo Y., Hara T., Katsuki T., Toyofuku A., Katsura Y., Takikawa O., Fujii T., Ohama K. (2004). Mechanisms regulating the expression of indoleamine 2,3-dioxygenase during decidualization of human endometrium. Hum. Reprod..

[69] Suzuki S., Toné S., Takikawa O., Kubo T., Kohno I., Minatogawa Y. (2001). Expression of indoleamine 2,3-dioxygenase and tryptophan 2,3-dioxygenase in early concepti. Biochem. J..

[70] Fukuwatari T., Murakami M., Ohta M., Kimura N., Jin-No Y., Sasaki R., Shibata K. (2004). Changes in the urinary excretion of the metabolites of the tryptophan-niacin pathway during pregnancy in Japanese women and rats. J. Nutr. Sci. Vitaminol..

[71] Stone T.W. (1993). Neuropharmacology of quinolinic and kynurenic acids. Pharmacol. Rev..

[72] Swartz K.J., During M.J., Freese A., Beal M.F. (1990). Cerebral synthesis and release of kynurenic acid: an endogenous antagonist of excitatory amino acid receptors. J. Neurosci..

[73] Sarsero J.P., Merino E., Yanofsky C. (2000). A *Bacillus subtilis* operon containing genes of unknown function senses tRNATrp charging and regulates expression of the genes of tryptophan biosynthesis. Proc. Natl. Acad. Sci. U.S.A..

[74] Sanni L.A., Thomas S.R., Tattam B.N., Moore D.E., Chaudhri G., Stocker R., Hunt N.H. (1998). Dramatic changes in oxidative tryptophan metabolism along the kynurenine pathway in experimental cerebral and noncerebral malaria. Am. J. Pathol..

[75] Burke F., Knowles R.G., East N., Balkwill F.R. (1995). The role of indoleamine 2,3-dioxygenase in the anti-tumour activity of human interferon-gamma *in vivo*. Int. J. Cancer.

[76] Moffett J.R., Namboodiri M.A. (2003). Tryptophan and the immune response. Immunol. Cell Biol..

[77] Terness P., Bauer T.M., Röse L., Dufter C., Watzlik A., Simon H., Opelz G. (2002). Inhibition of allogeneic T cell proliferation by indoleamine 2,3-dioxygenase–expressing dendritic cells: mediation of suppression by tryptophan metabolites. J. Exp. Med..

[78] Fallarino F., Grohmann U., Vacca C., Bianchi R., Orabona C., Spreca A., Fioretti M.C., Ouccetti P. (2002). T cell apoptosis by tryptophan catabolism. Cell Death Differ..

[79] Clark C.J., Mackay G.M., Smythe G.A., Bustamante S., Stone T.W., Phillips R.S. (2005). Prolonged survival of a murine model of cerebral malaria by kynurenine pathway inhibition. Infect. Immun..

[80] Badawy A.A.-B., Bano S., Steptoe A. (2011). Tryptophan in alcoholism treatment I: kynurenine metabolites inhibit the rat liver mitochondrial low k_m_ aldehyde dehydrogenase activity, elevate blood acetaldehyde concentration and induce aversion to alcohol. Alcohol Alcohol..

[81] Takikawa O., Yoshida R., Kido R., Hayaishi O. (1986). Tryptophan degradation in mice initiated by indoleamine 2,3-dioxygenase. J. Biol. Chem..

[82] Kolodziei L.R., Paleolog E.M., Williams R.O. (2011). Kynurenine metabolism in health and disease. Amino Acids.

[83] Fuchs D., Möller A.A., Reibnegger G., Werner E.R., Werner-Felmayer G., Dierich M.P., Wachter H. (1991). Increased endogenous interferon-gamma and neopterin correlate with increased degradation of tryptophan in human immunodeficiency virus type 1 infection. Immunol. Lett..

[84] Green A.R., Bloomfield M.R., Woods H.F., Seed M. (1978). Metabolism of an oral tryptophan load by women and evidence against the induction of tryptophan pyrrolase by oral contraceptives. Br. J. Clin. Pharmacol..

[85] Huether G., Hajak G., Reimer A., Poeggeler B., Blömer M., Rodenbeck A., Rüther E. (1992). The metabolic fate of infused tryptophan in men: possible clinical implications of the accumulation of circulating tryptophan and tryptophan metabolites. Psychopharmacology.

[86] Salter M., Bender D.A., Pogson C.I. (1985). Leucine and tryptophan metabolism in rats. Biochem. J..

[87] Badawy A.A.-B., Lake S.L., Dougherty D.M. (2014). Mechanisms of the pellagragenic effect of leucine: stimulation of hepatic tryptophan oxidation by administration of branched-chain amino acids to healthy volunteers and the role of free tryptophan and total kynurenines. Int. J. Tryptophan Res..

[88] Young S.N., Sourkes T.L. (1975). Tryptophan catabolism by tryptophan pyrrolase in rat liver: the effect of tryptophan loads and changes in tryptophan pyrrolase activity. J. Biol. Chem..

[89] Williams J.N., Feigelson P., Elvehjem C.A. (1950). Relation of tryptophan and niacin to pyridine nucleotides of tissue. J. Biol. Chem..

[90] Nishizuka Y., Hayaishi O. (1963). Studies on the biosynthesis of nicotinamide adenine nucleotides I. enzymatic synthesis of niacin ribonucleotides from 3-hydroxyanthranilic acid in mammalian tissue. J. Biol. Chem..

[91] Von Bubnoff D., Matz H., Frahnert C., Rao M.L., Hanau D., de la Salle H., Bieber T. (2002). FcepsilonR1 induces the tryptophan degradation pathway involved in regulating T cell responses. J. Immunol..

[92] Evans R.W., Powers R.W., Ness R.B., Cropcho L.J., Daftary A.R., Harger G.F., Vergona R., Finegold D.N. (2003). Maternal and fetal amino acid concentrations and fetal outcome during pre-eclampsia. Reproduction.

[93] Tanigushi K., Okatani Y., Sagara Y. (1994). Serotonin metabolism in fetus in preeclampsia. Asia Oceania J. Obstet. Gynaecol..

[94] Tanigushi K., Okatani Y., Sagara Y. (1990). Study on serotonin metabolism in toxemia of pregnancy. Nippon Sanka Fujinka Gakkai Zasshi.

[95] Horne C.H.W., Howie P.W., Goudie R.B. (1970). Serum alpha_2_-macroglobulin, transferrin, albumin and IgG levels in preeclampsia. J. Clin. Pathol..

[96] Benoit J., Rey E. (2011). Preeclampsia: should plasma albumin level be a criterion for severity. J. Obstet. Gynecol. Can..

[97] Villa P.M., Laivuori H., Kajantie E., Kajja R. (2009). Free fatty acid profiles in preeclampsia. Prostaglandins Leukot. Essent. Fatty Acids.

[98] Hubel C.A., Mclaughlin M.K., Evans R.W., Hauth B.A., Sims C.J., Roberts J.M. (1996). Fasting serum triglycerides, free fatty acids, and malonaldehyde are increased in preeclampsia, are positively correlated, and decrease within 48 hours postpartum. Am. J. Obstet. Gynecol..

[99] Endersen M.J.R., Morris J.M., Nobrega A.C., Buckley D., Linton E.A., Redman C.W.G. (1996). Serum from preeclamptic women induces vascular cell adhesion molecule-1 expression on human endothelial cells *in vitro*: A possible role of increased circulating levels of fatty acids. Am. J. Obstet. Gynecol..

[100] Catov J.M., Bertolet M., Chen Y.-F., Evans R.W., Hubel C.A. (2014). Non-esterified fatty acids and spontaneous pre-term birth: a factor analysis for identification of risk patterns. Am. J. Epidemiol..

[101] Kragh-Hansen U. (1991). Octanoate binding to the indole- and benzodiazepine-binding region of human serum albumin. Biochem. J..

[102] Bohney J.P., Feldhoff R.C. (1992). Effects of nonenzymatic glycosylation and fatty acids on tryptophan binding to human serum albumin. Biochem. Pharmacol..

[103] Curzon G., Friedel J., Knott P.J. (1973). The effect of fatty acids on the binding of tryptophan to plasma protein. Nature.

[104] Bernardi F., Guolo F., Bartolin T., Petronilho F., Dal-Pizzol F. (2008). Oxidative stress and inflammatory markers in normal pregnancy and preeclampsia. J. Obstet. Gynecol. Res..

[105] Sacks G.P., Studena K., Sargent K., Redan C.W. (1998). Normal pregnancy and preeclampsia both produce inflammatory changes in peripheral blood leukocytes akin to those of sepsis. Am. J. Obstet. Gynecol..

[106] Szarka A., Rigo J., Lazar L., Beko G., Molvarec A. (2010). Circulating cytokines, chemokines and adhesion molecules in normal pregnancy and preeclampsia determined by multiplex suspension array. BMC Immunol..

[107] Record C.O., Buxton B., Chase R.A., Curzon G., Murray-Lyon I.M., Williams R. (1976). Plasma and brain amino acids in fulminant hepatic failure and their relationship to hepatic encephalopathy. Eur. J. Clin. Invest..

[108] Hijikata Y., Hara K., Shiozaki Y., Murata K., Sameshima Y. (1984). Determination of free tryptophan in plasma and its clinical applications. J. Clin. Chem. Clin. Biochem..

[109] Hiratsuka C., Fukuwatari T., Sano M., Saito K., Sasaki S., Shibata K. (2013). Supplementing healthy women with up to 5.0 g/d of L-tryptophan has no adverse effects. J. Nutr..

[110] Dougherty D.M., Marsh-Richard D.M., Mathias C.W., Hood A.J., Addicott M.A., Moeller F.G., Morgan C.J., Badawy A.A.-B. (2008). Comparison of 50- and 100-g *L-*tryptophan depletion and loading formulations for altering 5-HT synthesis: pharmacokinetics, side effects and mood states. Psychopharmacology.

[111] Badawy A.A.-B., Dougherty D.M., Richard D.M. (2010). Specificity of the acute tryptophan and tyrosine plus phenylalanine depletion and loading tests Part II: normalisation of the tryptophan and tyrosine plus phenylalanine to competing amino acid ratios in a new control formulation. Int. J. Tryptophan Res..

[112] Schmidt S.K., Müller A., Heseler K., Woite C., Spekker K., MacKenzie C.R., Däubener W. (2009). Antimicrobial and immunoregulatory properties of human tryptophan 2,3-dioxygenase. Eur. J. Immunol..

[113] Müller A., Heseler K., Schmidt S.K., Spekker K., MacKenzie C.R., Däubener W. (2009). The missing link between indoleamine 2,3-dioxygenase mediated antibacterial and immunoregulatory effects. J. Cell. Mol. Med..

[114] Holtan S.G., Chen Y., Kaimal R., Creedon D.J., Enninga E.A. L., Nevala W.K., Markovic S.N. (2015). Growth modeling of the maternal cytokine milieu throughout normal pregnancy: macrophage-derived chemokine decreases as inflammation/counterregulation increases. J. Immunol. Res..

[115] Hannan N.J., Bambang K., Kaitu´u-Lino T.J., Konje J.C., Tong S. (2014). A bioplex analysis of cytokines and chemokines in first trimester plasma to screen for predictors of miscarriage. PLoS One.

[116] Yue C.-Y., Zhang B., Ying C.M. (2015). Elevated serum level of IL-35 associated with the maintenance of maternal-fetal immune tolerance in normal pregnancy. PLoS One.

[117] Curry A.E., Vogel I., Skogstrand K., Drewsa C., Schendel D.E., Flanders W.D., Hougaard D.M., Thorsen P. (2008). Maternal plasma cytokines in early- and mid-gestation of normal human pregnancy and their association with maternal factors. J. Reprod. Immunol..

[118] Ferguson K.K., McElrath T.F., Chen Y.-H., Mucherjee B., Meeker J.D. (2014). Longitudinal profiling of inflammatory cytokines and C-reactive protein during uncomplicated and preterm pregnancy. Am. J. Reprod. Immunol..

[119] Kraus T.A., Sperling R.S., Engel S.M., Lo Y., Kellerman L., Singh T., Loubeau M., Ge Y., Garrdo J.L., Rodriguez-Garcia M., Moran T.M. (2010). Peripheral blood cytokine profiling during pregnancy and post-partum periods. Am. J. Reprod. Immunol..

[120] Denney J.M., Nelson E.L., Wadhwa P.D., Waters T.P., Mathew L., Chung E.K., Goldenberg R.L., Culhane T.F. (2011). Longitudinal modulation of immune system cytokine profile during pregnancy. Cytokine.

[121] Burns C., Hall S.T., Smith R., Blackwell C. (2015). Cytokine levels in late pregnancy: are female infants better protected against inflammation?. Front. Immunol.

[122] Martínez-Varea A., Pellicer B., Perales-Marín A., Pellicer A. (2014). Relationship between maternal immunological response during pregnancy and onset of preeclampsia. J. Immunol. Res..

[123] Arriaga-Pizano L., Jimenez-Zamudio L., Vadillo-Ortega F., Martinez-Flores A., Herrerias-Canedo T., Hernandez-Guerrero C. (2005). The predominant Th1 cytokine profile in maternal plasma of preeclamptic women is not reflected in the choriodecidual and fetal compartments. J. Soc. Gynecol. Invest..

[124] Jonsson Y., Rubèr M., Matthiesen L., Berg G., Nieminen K., Sharma S., Ernerudh J., Ekerfelt C. (2006). Cytokine mapping of sera from women with preeclampsia and normal pregnancies. J. Reprod. Immunol..

[125] Laskowska M., Laskowska K., Leszczyńska-Gorzelak B., Oleszczuk J. (2007). Comparative analysis of the maternal and umbilical interleukin-8 levels in normal pregnancies and in pregnancies complicated by preeclampsia with intrauterine normal growth and intrauterine growth retardation. J. Matern. Fetal Neonatal Med..

[126] Tosun M., Celik H., Avci B., Yavuz E., Alper T., Malatyalioğlu E. (2010). Maternal and umbilical serum levels of interleukin-6, interleukin-8, and tumor necrosis factor-alpha in normal pregnancies and in pregnancies complicated by preeclampsia. J. Matern. Fetal Neonatal Med..

[127] Cembil Arikan D., Aral M., Coskun A., Ozer A. (2012). Plasma IL-4, IL-8, IL-12, interferon-γ and CRP levels in pregnant women with preeclampsia, and their relation with severity of disease and fetal birth weight. J. Matern. Fetal Neonatal Med..

[128] Dabagh-Gorjani F., Anvari F., Zolghadri J., Kamali-Sarvestani E., Gharesi-Fard B. (2014). Differences in the expression of TLRs and inflammatory cytokines in pre-eclamptic compared with healthy pregnant women. Iran J. Immunol..

[129] Svensson-Arvelund J., Mehta R.B., Lindau R., Mirrasekhian E., Rodriguez-Martinez H., Berg G., Lash G.E., Jenmalm M.C., Ernerudh J. (2015). The human fetal placenta promotes tolerance against the semiallogeneic fetus by inducing regulatory T cells and homeostatic M2 macrophages. J. Immunol..

[130] Lee S.M., Park J.S., Norwitz E.R., Oh S., Kim E.J., Lee J., Kim B.J., Park C.W., Jun K.J.K. (2015). Mid-trimester amniotic fluid pro-inflammatory biomarkers predict the risk of spontaneous preterm delivery in twins: a retrospective cohort study. J. Perinatol..

[131] Hernandez R.J., Manjarréz G.G., Chagoya G. (1989). Newborn humans and rats malnourished in utero: free plasma *L*-tryptophan, neutral amino acids and brain serotonin synthesis. Brain Res..

[132] Nilsen R.M., Bjørke-Monsen A.-L., Midttun O., Nygård O., Pedersen E.R., Ulvik A., Magnus P., Gjessing H.K., Vollset S.E., Ueland P.M. (2012). Materal tryptophan and kynurenine pathway metabolites and the risk of preeclampsia. Obstet. Gynecol..

[133] Løset M., Mundal S.B., Johnson M.P., Fenstad M.H., Freed K.A., Lian I.A., Eide I.P., Bjørge L., Blangero J., Moses E.K., Austgulen R. (2011). A transcriptional profile of the decidua in preeclampsia. Am. J. Obstet. Gynecol..

[134] Yusuf K., Kliman H.J. (2008). The fetus, not the mother, elicits maternal immunologic rejection: lessons from discordant dizygotic twin placentas. J. Perinat. Med..

[135] Badawy A.A.-B. (2014). Pellagra and alcoholism: a biochemical perspective. Alcohol Alcohol..

